# Basic Coagulation Parameters among Human Immunodeficiency Virus-Infected Adults in Gondar, Northwest Ethiopia: A Comparative Cross-Sectional Study

**DOI:** 10.1155/2018/5320827

**Published:** 2018-05-15

**Authors:** Masresha Seyoum, Bamlaku Enawgaw, Zegeye Getaneh, Getabalew Engidaye, Fikir Asrie, Mulugeta Melku

**Affiliations:** ^1^Department of Hematology and Immunohematology, School of Biomedical and Laboratory Sciences, College of Medicine and Health Sciences, University of Gondar, Gondar, Ethiopia; ^2^University of Gondar Referral Hospital Laboratory, University of Gondar, Gondar, Amhara Regional State, Ethiopia; ^3^Debre Birhan Referral Hospital Laboratory, Debre Birhan, North Shewa, Amhara Regional State, Ethiopia

## Abstract

**Objective:**

We aimed at assessing the basic coagulation parameters of HIV-infected adults at the University of Gondar Hospital antiretroviral therapy clinic.

**Methods:**

A comparative cross-sectional study was conducted from February to May 2017. A total of 300 study participants, consisting of 100 HAART-naïve HIV-infected adults, 100 HIV-infected adults who were taking HAART, and 100 HIV-seronegative apparently healthy adults, were included. Basic coagulation functional assays such as PT, APTT, and INR were determined by coagulation analyzer. CD4 cells and platelet count were analyzed by FACS count and SYSMEX K-21N automated analyzer, respectively. The data were entered, cleaned, and edited using Epi Info version 7 and analyzed using SPSS version 20. Kruskal-Wallis H, Dunn-Bonferroni pairwise comparison test, and Spearman's rank-order correlation analysis were used for inferential statistics. The results were expressed by a median and presented in tables. *P* value < 0.05 was considered as statistically significant.

**Results:**

PT, APTT, and INR were significantly higher, whereas platelet count was significantly lower in HIV-infected adults (both who were taking HAART and HAART-naïve) than HIV-seronegative adults (*P* < 0.001). PT and INR were significantly higher, and platelet count was significantly lower in HAART-naïve HIV-infected adults than HIV-infected adults who were taking HAART. In Spearman's rank-order correlation analysis, APTT has shown a significant negative correlation with a CD4 count in HAART-naïve HIV-infected adults.

**Conclusion:**

HIV-infected adults are more likely to develop coagulation abnormality than HIV-seronegative subjects. Coagulation parameters need to be checked regularly to monitor coagulation disorders in HIV-infected adults.

## 1. Introduction

Human immunodeficiency virus causes acquired immunodeficiency syndrome and causes significant morbidity and mortality by various mechanisms and one among them is hemostatic abnormalities [1, 2]. HIV continues to be a major global public health issue. As to World Health Organization (WHO) report of 2016, more than 35 million are living with HIV, of which about three-fourth are living in WHO African Region. Moreover, 1.0 and 1.8 million people died from HIV-related causes and became newly infected globally in 2016, respectively [3].

Hemostasis is a system that consists of different cellular and biochemical events that play a main role in keeping the blood in a liquid state within circulation, to prevent the occurrences of thrombotic and bleeding complication [4]. Different systems are working together to maintain the balance between excessive bleeding and thrombosis including a vascular system, coagulation system, fibrinolytic system, platelets, and protease inhibitors. Those systems work together to prevent bleeding and clotting problem [5]. In normal healthy individuals, the hemostatic system is in a delicate balance between excessive bleeding state and clotting. However, abnormalities in the system cause either hemorrhagic or clotting disorder [6].

There are different factors that affect the normal hemostatic system, of which HIV infection is known to have been one of the main causes of hemostatic abnormality [7]. HIV infection causes serious haemostatic complication especially in the late stage of HIV infection, as immune suppression, and the presence of concurrent infection or neoplastic diseases exacerbates the condition [8]. It is the major causes of the haematological disorder, as the virus deregulates haematopoiesis process and the coagulation system. The virus itself, virus-associated opportunistic infections, adverse effect of antiretroviral therapies (ART), and other associated complications were the possible cause of the abnormalities [9–11].

Coagulation abnormalities in HIV patients can be attributed from the effect of the virus which can cause a number of abnormalities that predispose the patients to the occurrences of coagulation disorder. The abnormalities caused by HIV infection include thrombocytopenia [11], endothelial cell dysfunction, activation of coagulation factors, and presence of anti-phospholipids antibody [12]. Both in vivo and in vitro studies showed that HIV has an ability to bind to the host cell via their receptor found on the surface of the host cells. For example, host cells that consist of the CD4 receptor, coreceptor chemokines ligand 4 (CXCR4), and chemokines receptor 5 (CCR5) interact with HIV, with the help of Glycoprotein 120 (gp120). This interaction reduces nitric oxide expression that results in endothelial cell dysfunction and causes the impaired immune function of the vascular endothelial cell [13, 14].

Endothelial cells synthesize different molecules that are involved in coagulation and fibrinolysis process. The component produced by endothelial cells includes von Willebrand factor (vWF), tissue plasminogen activator, plasminogen activator inhibitor, and protein S [15]. In HIV-infected individuals, endothelial dysfunction is a common complication caused by the effect of the virus and virus-associated immunological response. Such endothelial dysfunction activates the coagulation system, leading to the consumption of coagulation factors [16]. Moreover, HIV infection causes various abnormalities predisposing to a hypercoagulable state including the presence of antiphospholipid antibodies and lupus anticoagulant, deficiencies of protein C, protein S, heparin cofactor II, and antithrombin and increased levels of vWF and D-dimers [17, 18].

Persistent systematic inflammation, immune dysfunction, activation of lymphocyte and monocytes, and elevated level of inflammatory cytokine are the hallmarks of HIV infection, particularly in untreated and late stage of HIV infections [1]. Thus, inflammation modulates thrombotic response by upregulating procoagulants, downregulating anticoagulants, and suppressing fibrinolysis. The tissue damage, by either viral-mediated or immune-mediated mechanisms, releases cytokine and cellular products. These situations activate the endothelial surface to increase tissue factor expression, reduce endogenous anticoagulant signals, and promote leukocyte infiltration [19, 20].

HIV-associated thrombocytopenia limits the hemostatic role of platelet, particularly the primary hemostasis [21]. HIV infection can also cause non-acquired immune deficiency syndrome (AIDS) related complication including liver and renal diseases. Such complications limit the production of most coagulation factors, natural anticoagulants, fibrinolytic factors, and hematopoietic growth hormones which may contribute for hemostatic disorders [22, 23]. Furthermore, highly active antiretroviral therapy (HAART) drugs have adverse effects and cause hepatic damage that leads to a decreased production of coagulation factors [12]. HAART drugs especially protease inhibitors cause endothelial dysfunction by their effects on the metabolism of lipid and glucose [24]. Hence, the main aim of this study was to assess basic coagulation parameters of HIV-infected in comparison with HIV-seronegative individuals in Gondar, Northwest Ethiopia.

## 2. Materials and Methods

### 2.1. Study Design, Area, and Period

A comparative cross-sectional study was conducted at the University of Gondar Referral Hospital ART clinic from February to May 2017. The University of Gondar Referral Hospital is found in Gondar town, North Gondar zone, Amhara Regional State, Ethiopia. The town is situated at an altitude of 2133 meters above the sea level. According to the 2007 Ethiopian census report, the projectile population of Gondar town in 2015 are 323,900 [25]. Currently, the town has one referral hospital and five government health centres. The University of Gondar Referral Hospital is a teaching hospital which serves more than five million people in North Gondar zone and peoples of the neighbouring zones. The HIV care service of the hospital began in 2005, and it has three adult ART clinics, one paediatric, one Voluntary Counselling and Testing (VCT) clinic, and 2 adherence counselling clinics. Since 2005, when the hospital initiated ART, 7581 adults and 738 paediatrics patients have been enrolled. Currently, 5000 adults are actively being treated. On average, around 400 HIV-infected adults initiate HAART annually.

### 2.2. Source and Study Population

#### 2.2.1. Source Population

All HIV-infected adults who were attending the University of Gondar Referral Hospital ART clinic were taken as the source population for HIV-infected adult groups. For the healthy control group, all adults who were visiting VCT clinic were taken as the source population.

#### 2.2.2. Study Population

HIV-infected adults who were HAART-naïve and taking HAART at the University of Gondar Referral Hospital ART clinic during the study period were included in the study for the case groups. For healthy control, those serologically confirmed HIV-negative apparently healthy adults who were visiting VCT clinic during the study period were included in the study.

### 2.3. Inclusion and Exclusion Criteria

#### 2.3.1. Inclusion Criteria

For HIV-infected groups, serologically confirmed HIV-infected adults who were HAART-naïve and were taking HAART for at least one year were included in the study. For the healthy control group, serologically confirmed HIV-seronegative and apparently healthy adults who were volunteers to participate were included in the study.

#### 2.3.2. Exclusion Criteria

Pregnant women, adults who were on anticoagulant therapy, adults having a history of chronic disease like hypertension, cardiac disease, and diabetes mellitus, and adults who were positive for hepatitis B surface antigen test (HBsAg) and hepatitis C virus (HCV) were excluded from the study for all groups.

### 2.4. Sample Size and Sampling Techniques

According to rules of thumb that have been recommended by van Voorhis and Morgan, 30 participants per group are required to detect real differences, which could achieve an 80% power [26]. Thus, a total of 300 study participants (100 HAART-naïve HIV-infected adults, 100 HIV-infected adults who were taking HAART, and 100 HIV-seronegative apparently healthy age and sex-matched control) were enrolled in the study. A systematic random sampling technique was used to select study participants at the University of Gondar Referral Hospital ART clinic and VCT clinic.

### 2.5. Data Collection Procedure

Sociodemographic characteristics of study participant were collected using pretested structured questionnaire. The questionnaire consists of two parts. The first part of questionnaire consists of sociodemographic characteristics of study participants which include age, sex, marital status, and residence. The second part of questionnaire consists of questions regarding clinical information of study participants, HAART usage, duration of HAART, and WHO AIDS stage. Clinical information and physical diagnosis of HIV-seronegative adults were assessed by trained clinicians who were working at the University of Gondar Referral Hospital VCT clinic.

### 2.6. Laboratory Analysis

Eight millilitres of venous blood was collected: five millilitres in EDTA anticoagulated tube for CD4 cell count, platelet count, and serological tests; three millilitres in 3.2% sodium citrated anticoagulated tube for basic coagulation parameters analysis. Platelet count was determined using SYSMEX K-21N (Sysmex Corporation Kobe, Japan), a three-part differential automated haematological analyzer. CD4 positive cell count was determined using the FACS count automated analyzer (Becton Dickenson and Company, California, USA). For basic coagulation parameters (PT, aPTT, and INR), platelet poor plasma was prepared by centrifugation of sodium citrate anticoagulated blood at 1500 g for 15 minutes. Then, PT and APTT were determined using HUMACLOT DUE PLUS coagulation analyzer (Wiesbaden, Germany). HBsAg and HCV antibody were determined by colloidal gold enhanced immune-assay (Xiamen Fujian, China, for HBsAg; Xiamen Fujian, China, for HCV antibody). All laboratory tests were done following standard operating procedure and the manufacturers' recommendation.

### 2.7. Data Quality Management

The questionnaire was prepared in English, translated to local language-Amharic, and then translated back to English to check the consistency. Training was given for the data collectors about objective and relevance of the study, confidentiality issues, study participants' right, techniques of interview, and result recording. Standard operating procedures and manufacturer instructions were strictly followed throughout the procedures and all reagents were stored and prepared according to the manufacturer's instruction. The analytical quality of each laboratory method was checked by running control materials before analyzing the patients' samples. The analysis of patients' samples was done if and only if the methods passed the quality control checking.

### 2.8. Statistical Analysis

Data were coded and entered into Epi Info 7 statistical software and then exported to SPSS version 20 for analysis. Homogeneity of variance was checked using Levene's statistics. The Kolmogorov-Smirnov normality test was used for checking the distribution of continuous variables, and it revealed that the variables were not normally distributed for each group. Nonparametric tests, Kruskal-Wallis H, and Dunn-Bonferroni pairwise comparison were used for comparison of coagulation parameters between groups. Spearman's rank-order correlation analysis was used to assess the correlation of CD4 count with basic coagulation parameters and platelet count. The results were presented as median [interquartile range] (IQR). Tables were used to present the summarized data. In all statistical analysis, a *P* value < 0.05 was considered as statistically significant.

### 2.9. Ethical Consideration

The study was approved by School of Biomedical and Laboratory Sciences, the University of Gondar Research and Ethics Committee. Informed written consent was taken from each of the study participants. All the information obtained from the study participants was kept confidential. Laboratory test results were communicated to the responsible clinicians working at the University of Gondar Referral Hospital ART clinic and VCT clinic.

## 3. Results

### 3.1. General Characteristics of Study Participants

A total of 300 adults who were attending the University of Gondar Referral Hospital ART and VCT clinics from February to May 2017 were included in the study. The study subjects were categorized into three groups: apparently healthy HIV-seronegative adults; HIV-infected adults taking HAART; and HAART-naïve HIV-infected adults. Each group consisted of 100 individuals. The median age of the study subjects was 35 years (IQR: 15 year). Of these enrolled individuals, 53.3% (*n* = 160) were females. From those 200 HIV-infected adults, the majority of them were classified as WHO HIV/AIDS clinical stage I (Table 1).

### 3.2. Coagulation Profiles of Study Participants

As compared to apparently healthy HIV-seronegative group, PT, APTT, and INR were prolonged in HIV-infected adults who were HAART-naïve and taking HAART. For example, 10% apparently health HIV-seronegative adults, 62% HIV-infected adults who were taking HAART, and 87% of HIV-infected adults who were HAART-naïve had a prolonged PT value. Thrombocytopenia was found in 8% of HIV-infected adults who were taking HAART and 26% of HAART-naïve HIV-infected adults, but no thrombocytopenia was found in apparently healthy HIV-seronegative control group (Table 2).

In the apparently healthy control group, the median [IQR] values of PT, APTT, INR, platelet, and CD4 count were 13.6 [2.6] seconds, 28.0 [5.18] seconds, 1.16 [0.24], 269.0 [85.7] × 10^3^/*μ*l, and 629 [306] cells/*μ*l, respectively. The median [IQR] values of PT, APTT, INR, platelet, and CD4 count were 14.4 [2.57] seconds, 33.5 [10.22] seconds, 1.23 [0.25], 248 [83.0] × 10^3^/*μ*l, and 383 [201] cells/*μ*l for HIV-infected adults who were taking HAART, respectively (Table 3).

### 3.3. Comparison of Basic Coagulation Parameters among Study Groups

As the data were not normally distributed, nonparametric tests (Kruskal-Wallis H and Dunn-Bonferroni pairwise comparison test) were used to compare the median differences in basic coagulation parameters between study groups. In Kruskal-Wallis analysis, the median [IQR] of PT, APTT, and INR showed statistically significant difference among the study groups (*P* < 0.001). In multiple pairwise comparisons analysis using Dunn-Bonferroni pairwise comparison, the median [IQR] values of PT, APTT, and INR of HIV-infected adults (both HAART and HAART-naïve groups) were significantly higher than the control group (*P* < 0.05). The median [IQR] values of PT and INR were significantly prolonged in HAART-naïve HIV-infected adults compared to HIV-infected adults who were taking HAART (*P* < 0.001). But the median [IQR] value of APTT did not show significant differences between HAART-naïve and HAART groups (*P* = 0.201) (Table 3).

The platelet count was highest in apparently healthy control subjects and lowest in HAART-naïve HIV-infected adults. In Kruskal-Wallis analysis, the result showed that platelet count was significantly different among groups (*P* < 0.001). The median [IQR] of platelets was significantly lower in HIV-infected adults (both HAART-naïve and on-HAART) compared to apparently healthy control group (*P* < 0.05). Moreover, in Dunn-Bonferroni pairwise comparison test, the median [IQR] platelet count of HAART-naïve subjects was significantly lower compared to HIV-infected adults taking HAART (*P* < 0.001) (Table 3).

### 3.4. Correlation of CD4 Cell Count with Basic Coagulation Parameters in HIV-Infected Adults

The correlation between CD4 cell count and basic coagulation parameters (PT, APTT, INR, and platelet count) among pre-HAART and on-HAART HIV-infected adults is shown in Table 4. As indicated in Table 4, Spearman's rank-order correlation analysis showed that CD4 count had been significantly and negatively correlated with APTT in HAART-naïve HIV-infected adults (*rho* = −0.235; *P* = 0.018) (Table 4).

### 3.5. Comparison of Basic Coagulation Parameters Based on HAART Duration

In Kruskal-Wallis comparison, there were no statistically significant differences in the median [IQR] of PT, APTT, INR, and platelet count between HIV-infected adults taking HAART for <2 years, 2–5 years, and >5 years (*P* > 0.05) (Table 5).

## 4. Discussion

In the present study, the overall coagulation abnormality was higher in HIV-infected adults than HIV-seronegative individuals. Prolonged PT, APTT, INR, and low platelet count were found in HIV-infected adults. Different factors including HIV-associated endothelial dysfunction, the presence of anti-cardiolipin antibody, and liver disease were the possible reason for the occurrence of coagulation system abnormality in HIV patients [15, 27].

Our finding showed that platelet count was significantly decreased in HIV-infected adults (both on-HAART and HAART-naïve) compared to those HIV-seronegative adults (*P* value < 0.05). This study was in agreement with the researches done in Karimnagar, India, and Mbarara, Uganda, which found low platelet count in HIV patients compared to HIV negatives [12, 13]. Studies conducted in Sudan, Benin city of Nigeria, and the Anambra state of Nigeria also found low platelet count in HIV-infected individuals compared to healthy controls [28–30]. HIV infection reduces the platelets count via immune-mediated destruction of platelet and megakaryocytes, thrombotic thrombocytopenic purpura, impaired haematopoiesis [10, 31], and damaging of the liver [32]. However, in contrary to our study finding, a study conducted in Cape Coast, Ghana, found that there was no statistically significant difference in terms of platelet count between HIV-infected patients and healthy controls [33]. The discrepancy may be because of variation in population characteristics and male to female ratio. In the Ghanaian study, the numbers of female study participants in HIV-infected groups (75.5%) were higher than those in HIV-seronegative groups (8%). Evidence showed that the platelet count is higher in females than males [34]. As a result of this, the statistically significant difference in platelet count between the two groups might not be observed in Ghanaian study.

This study revealed that the platelet count was significantly reduced in HAART-naïve HIV-infected adults as compared to HIV-infected adults who were taking HAART (*P* < 0.001). Studies showed that HAART treatment reduces viral load and increases CD4 count and production of platelets [35]. The reduction of viral load and immune restoration in HIV-infected adults taking HAART may contribute for high platelet count as compared to HAART-naïve adults. Moreover, reduction of viral load may decrease HIV-associated hypercoagulative disorders [17].

In the present study, PT, APTT, and INR were significantly elevated in HIV-infected adults (both HAART and HAART-naïve) compared to HIV-seronegative adults (*P* value < 0.05). This finding was concordant with the studies done in Jimma town of Ethiopia, Abia state city of Nigeria, and Benin city of Nigeria which found significantly prolonged PT, APTT, and INR in HIV-infected individuals compared to controls [28, 36, 37]. Similarly, studies conducted in Owerri and Anambra also found significantly high PT and APTT in infected adults (*P* < 0.05) [7, 29]. HIV infection has been associated with endothelial dysfunction, liver disease, and factors predisposing for hypercoagulable state including the presence of antiphospholipid antibodies and lupus anticoagulant, and deficiencies of protein C and protein S which cause activation and consumption of coagulation factors might be the possible reason for the occurrence prolonged PT and APTT in HIV-infected individuals [15, 27, 38–40]. However, our finding was discordant with a study done in Sudan which did not find a significant difference in PT and APTT between HIV-infected individuals and healthy controls [41]. The possible reason for this variation may be due to differences in coagulation analyzers, variation in comparison method, and the use of minimum sample size in Sudanese study (only 40 study participants). But our study used relatively large sample size; thus it improves the power of the statistical model to show the real difference between the two groups.

The values of PT and INR value were significantly lower in HIV-infected adults who were taking HAART compared to HAART-naïve HIV-infected adults (*P* < 0.001). HAART has been strongly associated with restoration of CD4 cell count [35] and reduction of viral load, endothelial dysfunction, and abnormal coagulation activation [42]. Consequently, HAART may reduce the HIV-associated coagulation abnormalities, resulting in less prolongation of PT and INR. But this finding was discordant with the study done in Anambra state of Nigeria which finds no significant difference in PT value between HIV-infected adults who were taking HAART and HAART-naïve groups. The variation may be explained by the fact that a minimum of 6 months of HAART duration was considered for inclusion of HAART groups in Anambra study, whereas in our study a minimum of 1 year of HAART duration was considered as inclusion criteria in HAART group.

HAART treatment reduces the production of lupus anticoagulant, and thus HIV-infected individuals who were taking HAART have lower lupus anticoagulant than HAART-naïve subjects [43]. In this study, APTT value of HAART-naïve study subjects was higher than that of HAART groups, though the difference was not statistically significant (*P* = 0.074). Even if the result was not statistically significant, the high prevalence of lupus anticoagulant in HAART-naïve HIV-infected adults may be the possible reason for the occurrence of high APTT value in HAART-naïve subjects. Moreover, evidence suggested that HAART reduces the activation of inflammatory markers and thus normalizes the coagulation, though the concern that markers of endothelial activation (vWF), coagulation (APTT), and inflammation (fibrinogen) remained significantly elevated as compared to HIH-seronegative adults [44].

In Spearman's correlation test, the finding showed that CD4 count of HIV-infected adults who were taking HAART was not correlated with any coagulation parameters. But in HAART-naïve HIV-infected adults, the CD4 count was significantly and inversely correlated with APTT. The finding was in agreement with a study conducted in Nigeria, Iran, and India [12, 45, 46]. The possible reason is that when CD4 count decreases there was an increase in the incidence of lupus anticoagulant and anticardiolipin antibodies. Evidence revealed that 53.5% of HIV-infected adults had circulating anticoagulants [47]. Moreover, at the low CD4 count, HIV-associated endothelial activation and liver damage are common complications [48]. Endothelial activation and liver damage cause consumption of blood clotting factors and/or abnormal production of liver-dependent clotting factors that can result in the occurrence of prolonged APTT in HIV-infected adults [7].

Our finding indicated that HAART duration did not significantly differ between HIV-infected adults taking HAART for <2 years, 2–5 years, and >5 years (*P* > 0.05). This is in consonance with previous studies conducted in Anambra state of Nigeria which reported no significant difference of coagulation parameters among HAART experienced HIV-infected patients with regard to HAART duration [29].

## 5. Limitation of the Study

In this study, only basic coagulation parameters such as PT, APTT, INR, and platelet count were measured to assess coagulation profile. The study did not include factor assay, so it has a limitation in differentiating the exact cause of prolonged PT and APTT. Besides, measurement of inflammatory markers was no part of the study; this may influence the strength of statistical differences between study groups.

## 6. Conclusion

Coagulation abnormality was higher in HIV-infected adults than HIV-seronegative subjects. PT was significantly higher in HAART-naïve HIV-infected adults compared with an HIV-infected adults taking HAART subjects, whereas platelet count was significantly lower in HAART-naïve group compared to HAART group. In correlation analysis, only APTT was inversely correlated with a CD4 cell count in HAART-naïve HIV-infected adults. In our study, comparison of coagulation parameters between HAART-naïve and HAART groups indicated that HAART treatment has a positive impact on coagulation profiles of HIV-infected adults. Therefore, in addition to complete blood cell count, organ function test, and CD4 cell count, coagulation tests like PT and APTT need to be included in the routine tests for management of HIV-infected patients. Moreover, multicentred prospective studies by including the measurement of coagulation factors and inflammatory markers need to be conducted.

## Figures and Tables

**Table 1:** Sociodemographic and clinical characteristics of study participants at University of Gondar Hospital ART clinic and VCT unit (*n* = 300).

Variables	Study groups		
	Apparently healthy adults (*n* = 100)	HIV-infected adults who were taking HAART (*n* = 100)	HAART-naïve HIV infected adults (*n* = 100)
	*n* (%)	*n* (%)	*n* (%)
*Age (year)*			
18–24	39 (39)	3 (3)	7 (7)
25–34	34 (34)	21 (27)	35 (35)
35–44	16 (16)	41 (41)	31 (31)
45–54	8 (8)	27 (27)	16 (16)
>54	3 (3)	8 (8)	11 (11)

*Sex*			
Male	58 (58)	35 (35)	45 (45)
Female	42 (42)	65 (65)	55 (55)

*Residence*			
Urban	73 (73)	92 (92)	75 (75)
Rural	27 (27)	8 (8)	25 (25)

*Religion*			
Orthodox	93 (93)	90 (90)	88 (88)
Protestant	1 (1)	0	2 (2)
Muslim	6 (6)	8 (8)	9 (9)
Other	0	2 (2)	1 (1)

*Educational status*			
Unable to read and write	15 (15)	27 (27)	36 (36)
Primary school	24 (24)	16 (16)	27 (27)
Secondary school	31 (31)	37 (37)	23 (23)
Tertiary school	30 (30)	20 (20)	14 (14)

*Marital status *			
Single	67 (67)	10 (10)	30 (30)
Married	19 (19)	61 (61)	42 (42)
Divorced	11 (11)	8 (8)	18 (18)
Widowed	3 (3)	21 (21)	10 (10)

*WHO stage*			
Stage I		91 (91)	77 (77)
Stage II		8 (8)	13 (13)
Stage III		0	6 (6)
Stage IV		1 (1)	4 (4)

**Table 2:** Basic coagulation and platelet profiles of study participants.

Profile	HIV-seronegative adults (*n* = 100)	HIV-infected adults taking HAART (*n* = 100)	HAART-naïve HIV-infected adults (*n* = 100)	Reference range
	*n* (%)	*n* (%)	*n* (%)	
*PT*				
Normal	90 (90)	38 (38)	13 (13)	10–14 seconds
Prolonged	10 (10)	62 (62)	87 (87)	

*APPT*				
Normal	91 (91)	68 (68)	60 (60)	24–36 seconds
Prolonged	9 (9)	32 (32)	40 (40)	

*INR*				
Normal	90 (90)	44 (44)	15 (15)	0.8–1.2
Prolonged	10 (10)	56 (56)	85 (85)	

*Platelet count*				
Normal	100 (100)	92 (92)	74 (74)	150–400 × 10^3^/ul
Low	0	8 (8)	26 (26)	

HAART: highly active antiretroviral therapy; PT: prothrombin time; INR: international normalization ratio; APTT: activated partial thromboplastin time.

**Table 3:** Comparison of basic coagulation parameters, platelet count, and CD4 cells among groups (Kruskal-Wallis test and multiple pairwise comparison using Dunn-Bonferroni correction).

Parameters	HIV-seronegative adults (*n* = 100)	HIV-infected adults taking HAART (*n* = 100)	HAART-naïve HIV-infected adults (*n* = 100)	*P* value
	Median [IQR]	Median [IQR]	Median [IQR]	
PT (second)	13.6 [2.6]	14.4 [2.57]	15.8 [2.70]	<0.001^**∗**^
APPT (Second)	28 [5.18]	33.5 [10.22]	35 [11.6]	<0.001^**∗**^
INR	1.16 [0.24]	1.23 [0.25]	1.37 [0.28]	<0.001^**∗**^
Platelet count (×10^3^/*μ*l)	269 [85.7]	248 [83.0]	191 [104]	<0.001^**∗**^
CD4 count (Cells/*μ*l)	629 [306]	383 [201]	258 [170]	<0.001^**∗**^
Multiple pairwise comparisons using Dunn-Bonferroni correction				
Parameters	^a^Vs^b^	^a^Vs^c^	^b^Vs^c^	
PT (seconds)	0.011^*∗*^	<0.001^*∗*^	<0.001^*∗*^	
APTT (seconds)	<0.001^*∗*^	<0.001^*∗*^	0.201	
INR	<0.022^*∗*^	<0.001^*∗*^	<0.001^*∗*^	
Platelet count (×10^3^/*μ*l)	0.032^*∗*^	<0.001^*∗*^	<0.001^*∗*^	
CD4 count (Cells/*μ*l)	<0.001^*∗*^	<0.001^*∗*^	<0.001^*∗*^	

HAART: highly active antiretroviral therapy; PT: prothrombin time; INR: international normalization ratio; APTT: activated partial thromboplastin time; IQR: interquartile range; ^a^apparently healthy HIV-seronegative adults; ^b^HIV-infected adults who were taking HAART; ^c^HIV-infected adults who were HAART-naïve; ^*∗*^significant at *P* < 0.05.

**Table 4:** Spearman's rank-order correlation *(rho)* of CD4 cell count with basic coagulation parameters and platelet count among HIV-infected adults.

Groups of study participants	Variables	Correlation coefficient	Significance level
		*(rho)*	
HAART-naïve group (*n* = 100)	PT	−0.051	0.616
	APTT	−0.235	0.018^*∗*^
	INR	−0.078	0.438
	Platelet count	0.073	0.469

HAART group (*n* = 100)	PT	0.11	0.276
	APTT	−0.116	0.355
	INR	0.051	0.616
	Platelet count	0.095	0.346

^*∗*^Significant at *P* < 0.05; PT: prothrombin time; APTT: activated partial thromboplastin time; INR: international normalization ratio.

**Table 5:** Comparison of basic coagulation parameters and platelet count among HIV-infected adult taking HAART on the basis of HAART duration (Kruskal-Wallis test).

Parameters	Duration of HAART (*n* = 100)			Significance value
	<2 years	2–5 years	5 years	
	Median [IQR]	Median [IQR]	Median [IQR]	
PT	14.3 [3.15]	13.9 [2.05]	14.5 [2.75]	0.799
APTT	34.4 [7.30]	33.5 [11.95]	33.5 [12.08]	0.954
INR	1.22 [0.27]	1.18 [0.22]	1.24 [0.26]	0.778
Platelet count	253 [83.0]	253 [94.5]	238 [84.2]	0.774

HAART: highly active antiretroviral therapy; PT: prothrombin time; APTT: activated partial thromboplastin time; INR: international normalization ratio; IQR: interquartile range.

## Data Availability

Data will be available upon request from the corresponding author.

## Abbreviations

AIDS: Acquired immune deficiency syndrome
APTT: Activated partial thromboplastin time
ART: Antiretroviral therapy
BD: Becton Dickenson
CD: Cluster of differentiation
EDTA: Ethylene diamine tetra-acetate
FACS: Fluorescence-activated cell sorting
HAART: Highly active antiretroviral therapy
HBsAG: Hepatitis B surface antigen
HCV: Hepatitis C virus
HIV: Human immunodeficiency virus
IQR: Interquartile range
INR: International normalization ratio
PT: Prothrombin time
SPSS: Statistical Package for Social Science
VCT: Voluntary counselling test
vWF: von Willebrand factor.
